# Distribution of monetary incentives in health insurance scheme influences acupuncture treatment choices: An experimental study

**DOI:** 10.1371/journal.pone.0218154

**Published:** 2019-06-10

**Authors:** Ye-Seul Lee, Song-Yi Kim, Younbyoung Chae

**Affiliations:** 1 Acupuncture and Meridian Science Research Center, College of Korean Medicine, Kyung Hee University, Seoul, Korea; 2 Department of Anatomy and Acupoint, College of Korean Medicine, Gachon University, Seongnam, Korea; University of Ghana College of Health Sciences, GHANA

## Abstract

**Background:**

Understanding how doctors respond to occupational and monetary incentives in health care payment systems is important for determining the effectiveness of such systems. This study examined changes in doctors’ behaviors in response to monetary incentives within health care payment systems in a *ceteris paribus* setting.

**Methods:**

An online experiment was developed to analyze the effect of monetary incentives similar to fee-for-service (FFS) and capitation (CAP) on doctors’ prescription patterns. In the first session, no monetary values were presented. In the second session conducted 1 week later, doctors were randomly assigned to one of two monetary incentive groups (FFS group: n = 25, CAP group: n = 25). In all sessions, doctors were presented with 10 cases and asked to determine the type and number of treatments.

**Results:**

In the first session with no monetary incentives, there was no significant difference between the FFS and CAP groups in the number of treatments. When monetary incentives were provided, doctors in the CAP group prescribed fewer treatments than the FFS group. The perceived severity of the cases did not change significantly between sessions and between groups. linear mixed-effects regression model indicated the treatment choices were influenced by monetary incentives, but not by the perceived severity of the patient’s symptoms.

**Conclusion:**

The monetary values incentivized the doctors’ treatment choices, but not their professional evaluation of patients. Monetary values designed within health care systems influence the doctor’s decisions in the form of external rewards, in addition to occupational values, and can thus be adjusted by more effective incentives.

## Introduction

*“… I will follow that system of regimen which*, *according to my ability and judgment*, *I consider for the benefit of my patients*, *and abstain from whatever is deleterious and mischievous*.*”*–Hippocratic Oath

The ultimate occupational objective of doctors, i.e., to treat patients in the way they consider most beneficial, is systemized in the clinical setting within health policies, such as health care payment schemes [1]. The treatments that doctors provide to patients are not only curative for the patients, but also serve as a source of income for the doctors [2]. This relationship is represented by the agency relationship between doctors and patients, as well as the agency relationship between doctors and the health care system, in which the doctors’ medical decisions are linked to the execution of necessary public health measures [3, 4]. Previous studies have examined the nature of the occupational role of doctors and the effect of the value of the treatment as a prospective reward for their performance [1, 2, 5, 6]. Retrospective payment schemes, such as fee-for-service (FFS), encourage doctors to provide more services, while prospective payment schemes, such as capitation (CAP), reduce the provision of medical services [1–3, 6, 7]. This difference may be in conflict with the occupational objective of doctors, leading to arguments pertaining to moral hazard [1, 5]. Doctors’ behaviors appear to depend on the effectiveness of the health system in which they operate [8, 9].

Examining the influence of specific payment schemes on doctors’ medical decisions is difficult, and the responses are ex ante unknown. Empirical and theoretical studies have observed changes in doctors’ behaviors under various payment schemes [10–14]. Other studies suggest a mixed effect of payment system on doctors’ behaviors [15, 16]. Experimental studies have investigated altruistic or other-regarding behaviors of health care providers, and the effect of payment systems on different groups [2–4, 6, 7, 17]. The occupational goal of doctors, i.e., to provide optimal treatment, is affected by the extrinsic factor of monetary incentives [3, 17, 18]. Despite efforts to understand behavior under different incentive schemes, the heterogeneity of doctors’ intrinsic motivations and differences in health care systems makes it difficult to determine the causal effects of monetary incentives [6, 15].

The main health care reimbursement system in South Korea is a regulated form of FFS since the beginning of the national health insurance [19], and within this health care system, both Western and Korean Medicine is officially incorporated in which the health care providers are reimbursed by the regulated FFS [20]. Through the national health insurance, Korea gradually achieved nationwide health insurance covering its whole population in 1989, and the universal health coverage has been sustained to date [21]. On the other hand, as the aforementioned studies have pointed out, the payment scheme is an essential element of the monetary incentives of health care providers and is a key factor affecting the behavior of the doctors. Previous studies showed that the fee-for-service system in the national health insurance of Korea has led to an increase in both volume and intensity of medical services with a greater margin in the provision of services [19, 22]. As a response, there is a movement in the Korean government for a transition of health care reimbursement system from FFS to a prospective payment system [19].

**Fig 1:**
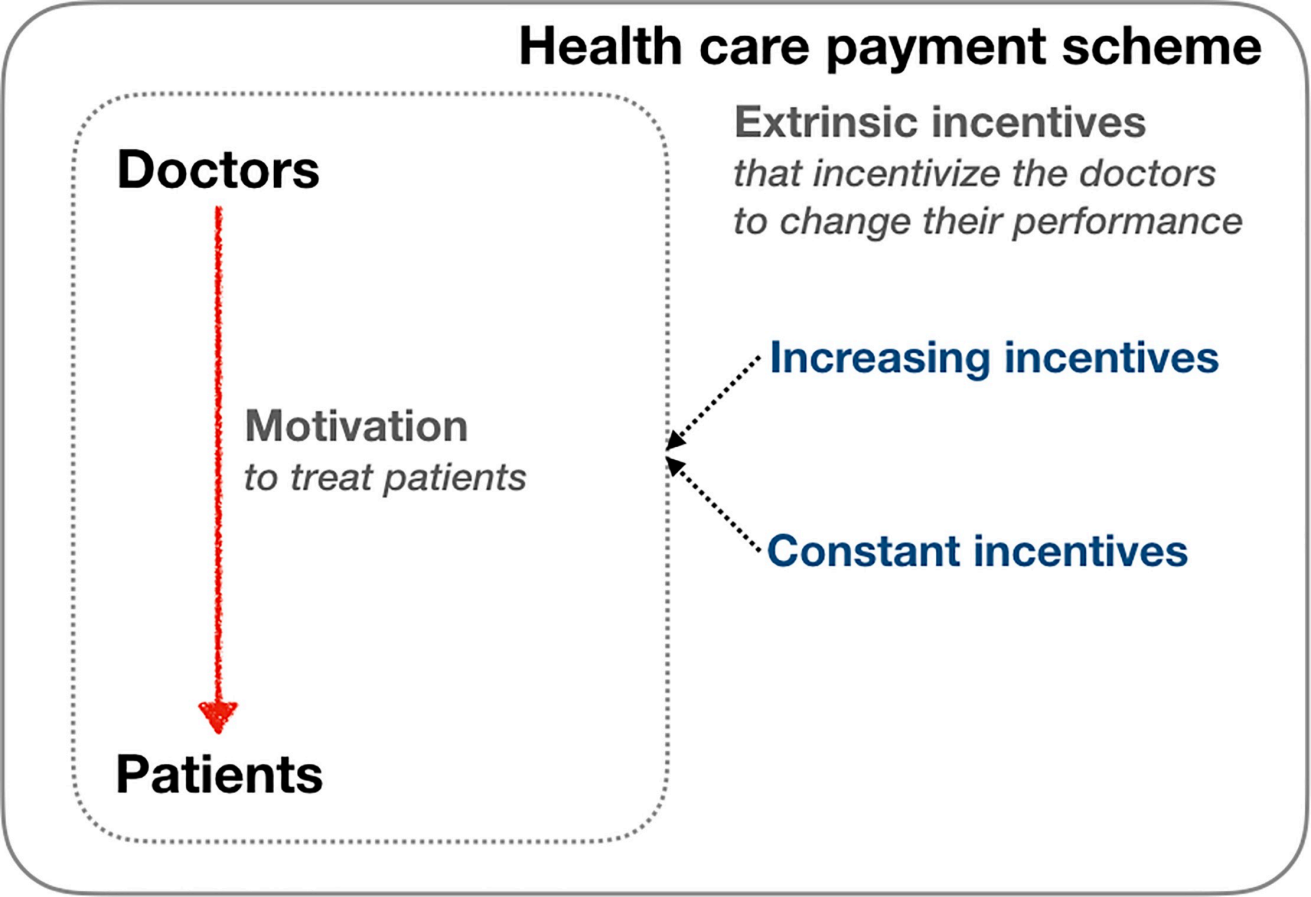
Hypothesis and study design. Doctors are motivated to treat patients, and health care payment schemes provide monetary incentives that may influence doctor behaviors.

This study evaluated changes in doctors’ decision-making in response to different forms of monetary incentives in a *ceteris paribus* setting. An online experiment was conducted, in which doctors participated in a virtual diagnosis and treatment prescription process that resembled an actual medical encounter. Our hypothesis was that doctors would implicitly respond to extrinsic monetary incentives, as reflected in the quantity of treatments prescribed, even without any explicit instruction related to their profit, and regardless of their intrinsic motivation to treat patients to the best of their knowledge (Fig 1). The study was structured as a two-part experiment in which doctors made decisions with and without monetary incentives.

## Methods

### Experimental design

An online experiment was developed with virtual diagnosis and treatment prescription components. Korean medical doctors with current clinical practices participated in two sessions with a 1-week interval. The characteristics of the participants are provided in Table 1 [23]. In an online setting, the purpose of the experiment was introduced as a project to collect prescription data from clinicians, and the doctors were asked to diagnose and prescribe the patient as they would in clinical practice based on the information provided on vignettes. While the doctors were tasked with diagnosing 10 cases and determining the medical services required for each case, the real purpose of the study was to determine the influence of monetary incentives on doctors’ prescription patterns. The experiment protocol carried out to the participating doctors were identical, and the participants could not proceed to the next step of the experiment if they did not complete each task. The detailed description of the experiment content is provided in Fig 1. In the first session, there were no monetary incentives. In the second session, doctors were randomized to two incentive groups using “randomize” function in R software (ver. 3.4.2 –“Short Summer”, http://r-project.org/), considering the possible confounders such as age, sex, years of clinical practice, type of medical institution in which the doctors were employed, and employment status. The monetary incentives presented to each group were similar to FFS or proportionally increasing monetary income (FFS group), or to CAP or constant monetary income per patient (CAP group). Both sessions presented 10 different cases with varying symptoms and diagnoses. The presentation of the patient’s cases, monetary incentives, and options of treatments were presented using Google Forms (gsuite.google.com/forms), and the data were collected in a .csv format. Outcome measures included the number of treatments prescribed and severity of the symptoms in each case. Written informed consent was obtained prior to participation. All procedures were conducted in accordance with the guidelines issued by the Human Subjects Committee and approved by the Institutional Review Board of Kyung Hee University, Seoul, Republic of Korea (KHSIRB-17-046).

**Table 1:** Characteristics of the participating doctors. FFS group: group presented with proportionally increasing monetary income; CAP group: group presented with constant monetary income per patient (CAP group).

		FFS group (n = 25)	CAP group (n = 25)	p-value
**Age (y)**	**20–29**	8	12	0.431
	**30–39**	13	12	
	**40–49**	3	1	
	**50+**	1	0	
**Sex**	**Male**	14	18	0.377
	**Female**	11	7	
**Experience (y)**	**1–4**	9	12	0.474
	**5–9**	11	11	
	**10+**	5	2	
**Medical institution**	**Public medical clinic**	6	4	0.885
	**Private clinic**	11	11	
	**Hospital**	7	9	
	**(Korean Medicine and Western Medicine)**			
	**Nursing home**	1	1	
**Employment status**	**Paid by the institution**	20	20	1
	**Operates own institution**	5	5	

### Participants

Doctors of Korean Medicine (n = 50) were recruited to participate in the online experiment, entitled: “A study on the acupuncture treatment patterns in Korean Medicine.” The minimum sample size was calculated a priori to the experiment for a medium effect size of 0.5, α error probability of 0.05, power of 0.8, and with two groups and two conditions, which yielded a total sample size of 34. Considering possible data loss, our initial goal for sample size was 40. Recruitment was conducted through online communities of doctors. Exclusion criteria included doctors who were not currently practicing in a medical institution, and those who did not use acupuncture as their primary treatment intervention. The anonymity of the doctors was maintained throughout the study. The data collected included age, sex, years of clinical practice, type of medical institution in which the doctors were employed, and employment status.

### Experimental procedures

The experiment used a two-session design with a 1-week interval, a modified version of study design from our previous study [24]. Instructions for both sessions stated that the purpose of the study was to analyze acupuncture treatment patterns based on actual patient data. No information with respect to monetary rewards was provided. Doctors were tasked with engaging in the same medical decision-making process that they would use in actual practice, where the cases presented were of actual patients. Ten cases with patient information were given, which were selected from among actual cases reports extracted from Korean medical hospital and clinic records (2014–2016). Patient information included primary symptoms, laboratory test results, radiology images if available, and patient history. The same cases were used in both sessions of the study.

Doctors were instructed to determine the severity of the symptoms using a numeric rating scale (NRS) ranging from 0 to 10 (0, not at all severe; 10, as severe as one can imagine). NRS was chosen to assess the symptom severity due to its usefulness across a wide range of symptoms assessment scales [25–27]. Using NRS as the unified measurement of symptom severity for all cases allowed for a comparison across cases as well as across doctors. Doctors then identified symptom patterns and determined the most appropriate combination of acupoints for treatment. Finally, the doctors indicated the treatment that they would prescribe to each patient based on their medical knowledge. These treatments included different types of acupuncture, moxibustion, cupping therapy, and physical therapies, all of which are covered by the National Health Insurance System (NHIS) of Korea.

**Fig 2:**
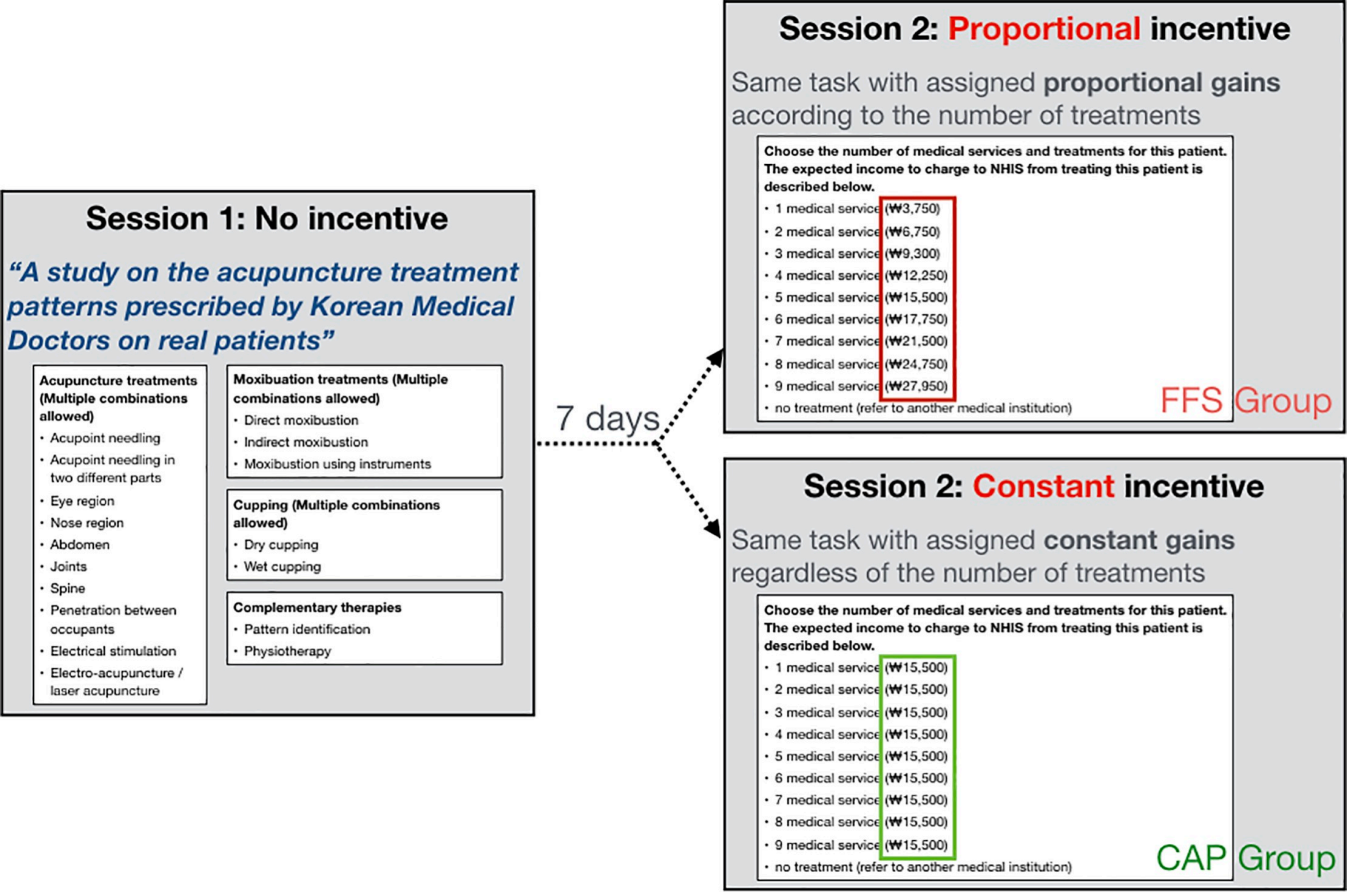
Experimental design. The doctors were instructed to indicate the treatments that they believed the patients needed. In Session 1, no monetary incentives were offered. Session 2 provided monetary rewards according to the number of treatments prescribed. The fee-for-service (FFS) group received typical FFS monetary incentives, and the capitation (CAP) group received typical CAP monetary incentives.

Instructions throughout the experiment were consistent, i.e., the doctors were instructed go through the medical process of diagnosis, treatment decision, and prescription. However, Session 2 presented additional information on monetary rewards without explicit instructions. Prior to deciding the specific treatments, the doctors were asked to identify the number of treatments to prescribe for each patient. In this step of Session 2, the estimated monetary reward was presented by each number. The doctors were uninformed of the additional information during the experiment when deciding on the number of treatments to prescribe, to conceal the real purpose of the study. In Session 2, doctors were divided into two groups which differed in the monetary gains achieved and expected profits, in accordance with the number of treatments prescribed. The FFS group was presented with monetary incentives that increased in proportion to the number of treatments prescribed. The CAP group was presented with monetary incentives that remained consistent regardless of the number of treatments prescribed (Fig 2). The only information provided to the participants, however, was that the aim was to collect data on medical diagnoses and treatments, and the information not provided to the participants were that monetary rewards appeared only in the second session and that there were two different types of monetary rewards.

### Statistical analysis

A two-way analysis of variance (ANOVA) was conducted to determine if there was a significant difference in the number of treatments prescribed according to the presentation of monetary rewards, between sessions (Session 1 and Session 2) and groups (FFS and CAP). A two-way ANOVA was conducted to determine whether there was a significant difference in the estimated symptom severity or NRS completed by the doctors according to the presentation of monetary rewards, between sessions (Session 1 and Session 2) and groups (FFS and CAP). To analyze the effects of monetary incentives and symptom severity on the number of treatments prescribed while controlling for doctor and patient characteristics, a linear mixed-effects regression model was fitted in which the participating doctors and cases were random intercepts. All statistical analyses were conducted using R software (ver. 3.4.2 –“Short Summer”, http://r-project.org/). A *p* value < 0.05 was considered statistically significant.

## Results

### Participant characteristics

A total of 50 doctors of Korean Medicine (FFS Group. n = 25; CAP Group. n = 25) participated in this study (average age = 31.66 ± 5.43 years; 32 males). Doctors worked in a variety of medical institutions, including public and private clinics, hospitals, and nursing homes. Most participants were employed by the medical institutions rather than running their own clinics. There were no significant differences in age, sex, years of clinical experience, type of medical institution, or employment status between the FFS and CAP groups (Table 1).

### Effects of health care payment schemes on doctors’ behaviors

#### Monetary incentives

**Fig 3:**
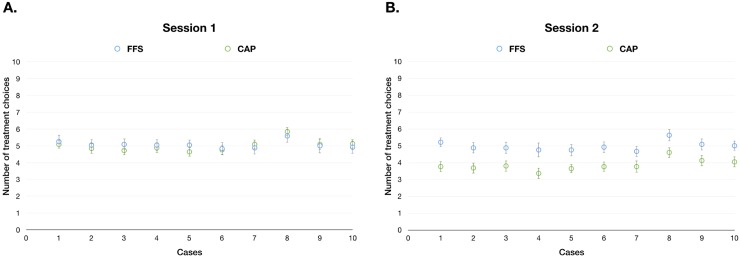
**A:** Number of treatments prescribed in the FFS and CAP groups in Session 1. **B:** Number of treatments prescribed in the FFS and CAP groups in Session 2. Two-way analysis of variance (ANOVA) showed significant differences in the number of treatments prescribed (main effect: F = 6.60, p = 0.049; between FFS and CAP: F = 6.082, p = 0.015; between Sessions 1 and 2: F = 4.719, p = 0.032). X-axis: case number; Y axis: number of treatments prescribed.

In Session 1 (no monetary incentive), there were no significant differences in the number of treatments prescribed between the FFS and CAP groups (Fig 3A). In Session 2, when monetary incentives were presented, doctors in the CAP group provided fewer treatments than those in the FFS group (Fig 3B). Two-way ANOVA indicated that the interaction effect (Session ˟ Group) was significant (main effect: F = 6.60, p = 0.049). The Session and Group effects were both significant (Group effect: F = 6.082, p = 0.015; Session effect: F = 4.719, p = 0.032).

#### Monetary incentives and symptom severity

**Fig 4:**
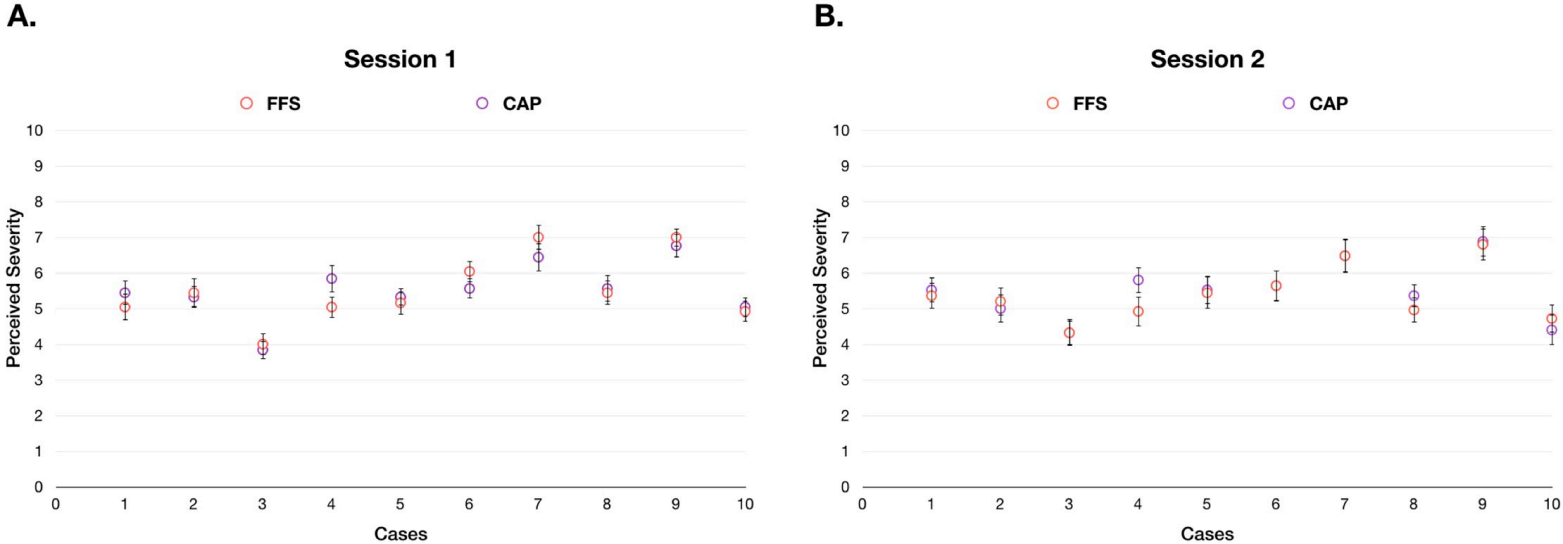
**A:** Perceived severity of symptoms in each case in the FFS and CAP groups in Session 1. **B:** Perceived severity of symptoms in each case in the FFS and CAP groups in Session 2. Two-way ANOVA revealed no significant differences in perceived symptom severity between groups or sessions (main effect: F = 0.596, p = 0.442; between FFS and CAP: F = 0.299, p = 0.586; between Sessions 1 and 2: F = 1.534, p = 0.219. X-axis: case number; Y axis: symptom severity as evaluated by the doctors.

There was no significant difference in perceived severity of symptoms between the FFS and CAP groups in Session 1 (Fig 4A). Additionally, there was no difference in perceived severity between the groups in Session 2 after the presentation of monetary incentives (Fig 4B). Two-way ANOVA indicated no significant difference in perceived symptom severity, between groups or sessions (between FFS and CAP: F = 0.299, p = 0.586; between Sessions 1 and 2: F = 1.534, p = 0.219; interaction: F = 0.596, p = 0.442).

#### Linear mixed-effects regression model

A linear mixed-effects regression model controlling for individual differences among doctors and cases (as random effects) was created (Table 2). The number of treatments was significantly affected by monetary incentives (β = -0.63, p < 0.001), but not by the perceived severity of patient symptoms (β = 0.02, p = 0.309). The interaction between monetary incentives and group was significant with respect to the change in the number of treatments prescribed (β = -1.04, p < 0.001), indicating that the monetary incentives affected the number of treatments according to group (FFS vs. CAP).

**Table 2:** Regression coefficients (β), confidence intervals (CI) and p-value for each fixed effect, and variability of the effects (σ^2^) from linear mixed-effects regression model.

	Number of treatments		
*Predictors*	*Estimates (β)*	*CI*	*p*
(Intercept)	4.96	3.88 – 6.05	**<0.001**
Monetary incentives	0.93	0.51 – 1.34	**<0.001**
Perceived symptom severity	0.02	-0.02 – 0.07	0.309
Groups	-0.05	-0.70 – 0.61	0.89
Groups:Monetary incentives	-1.04	-1.30 – -0.77	**<0.001**
***Random Effects***			
σ^2^	1.11		

## Discussion

In this study, doctors in clinical practice made virtual diagnoses and prescribed treatments through an online experiment. The results demonstrated that monetary incentives provided an incentive that influenced doctors’ treatment choices, in addition to their occupational motivation to treat patients to the best of their knowledge. Doctors prescribed fewer treatments when they were incentivized with a consistent monetary reward, regardless of their occupational motivations. These results contribute to current knowledge of the effect of health care payment schemes, which indicate that patients are overserved under FFS and underserved under CAP [2, 3, 7, 9, 14]. By demonstrating changed patterns of treatment decisions in response to monetary incentives, combined with the consistent ratings of symptoms severity evaluated by the doctors, causals could be drawn via a mixed model. The decision-making behavior of doctors was found to be influenced by monetary incentives but not by their evaluations of the symptom severity.

From a psychological perspective, Session 1 of the study tested the doctors’ occupational motivation to treat patients, which is related to altruistic behavior, empathy and agency [28–30]. Empathy is also related to social reputation and prosocial behavior [31–33]. On the other hand, Session 2 of the study tested doctors’ responses to monetary rewards. Estimated rewards act as incentives, and reduced rewards may decrease the motivation to undertake tasks when the level of effort required is held constant [18, 34]. Previous studies indicated that rewards can increase or reduce a person’s motivation to engage in a task [35]. Additionally, prosocial behavior is a mixture of responses to both external and intrinsic pressures and rewards [36]. We have previously reported a study on the general behavior of sharing pain in return for monetary rewards, in which the results showed that the distribution of rewards influence the number of shared pain [24]. It is noteworthy that, in our study, decision-making was not related to doctors’ evaluations of the symptom severity of the patients. While occupational goals play a crucial role in determining the decisions made by doctors [3, 17], payment schemes vary in the rewards that they provide. This leads to dynamic relationships among patients, doctors, and the health care systems in which the doctors mediate both the health care system and the patients, or play the role of agents between the two principals [37, 38].

In our study, there was no difference in the number of treatments prescribed by the FFS group between Sessions 1 and 2. While this can be interpreted in terms of positive reinforcement overcoming negative reinforcement, it may also be attributed to the doctors’ familiarity with the health care payment system in Korea. The main health care reimbursement system in Korea is FFS, which may explain similar behaviors between Sessions 1 (no monetary incentive) and 2 (monetary incentive) [19]. It should be interpreted with caution, therefore, to assume that fee-for-service leads to the same quantity of the treatment service compared with decisions with no rewards involved. The same approach implemented in other health care systems could lead to different results.

Incentive and reward schemes in medical settings, when effectively designed and implemented, could improve doctors’ performance and patient experience. While the interactions between doctors and patients have been understood from a societal perspective [39], the results of this study indicate that the structure of health care systems significantly affects both actors. Changes in doctor behavior may be mediated by moral hazard, but are also a product of external rewards, i.e., payments, and can thus be adjusted by more effective incentives. A system in which doctors receive monetary rewards when they provide the most effective treatments to patients, and in which patients are guaranteed to receive appropriate treatments without the requirement for exorbitant payments, may create a more effective and rewarding treatment environment.

This study introduces a novel approach in understanding the doctor’s behavior regarding treatment choices by introducing patient vignettes to practicing clinicians. From this study we were able to collect data of the doctor’s behavior while controlling for other factors such as patient differences. Previous studies investigated the possibility of patient vignettes in measuring health worker knowledge [40] and evaluating the effect of clinical decision support systems for improving hospital discharge decision-making [41, 42]. Furthermore, the concealment of the real purpose of the study and presenting the aim of the experiment to the doctors as a project to collect prescription data allowed this study to avoid strategic response biases by the doctors who might oppose payment schemes that disconnects the remuneration of doctors from the volume of medical service. By asking the doctors to diagnose and prescribe the patient as they would in clinical practice based on the information provided on vignettes, we were able to divert the doctors’ attention from the real purpose of the study and avoid possible response biases. Experimental and behavioral research in health care allows a *ceteris paribus* condition, which helps understand the participants’ behavior while other factors are controlled. This reduces bias and allows thorough robustness checks as researchers can repeat the experiment under the same setting [42].

The limits of our study must also be noted. One limitation of this study included the fact that the study simulated treatment prescriptions, and thus did not involve actual delivery of treatments to patients. Thus, it was not possible to evaluate the efficacy of the treatments. Studies have demonstrated quality-quantity tradeoffs between CAP and FFS [3, 6]. However, other studies employing an empirical approach have not demonstrated the same tradeoff between CAP and FFS [43]. Thus, further study is required to investigate the relationship between the efficacy of treatments and the health care payment system. Another limitation is the perceived symptoms severity measurement in our study, which was done by using NRS. While NRS is a simple and straightforward measurement for measuring symptom severity, it may lack in detail to encompass the severity evaluation in each case. In addition, the results of our study must be interpreted with caution for the lack of sample size to represent the whole population of doctors in the country. Finally, the recent introduction of pay-for-performance schemes may also require an experimental evaluation, i.e., of how monetary incentives affect the behavior of health care providers [44]. The results of this study may not be generalizable to reward-seeking behavior and aversion to negative reinforcement in non-medical settings. Additional studies are needed to understand the relationship between other-regarding and reward-seeking behavior.

This study demonstrates that extrinsic rewards delivered via healthcare payment systems act as incentives for doctors. Reduction in reward led to fewer prescriptions, even though doctors’ evaluations of patient symptoms remained unchanged. Doctors’ agency to patients, which manifests as altruistic behavior, may be affected by their agency to the health care system under different monetary incentive schemes. In addition to the contribution to current knowledge regarding the effect of health care payment system to the doctors’ behaviors, this study suggests a new approach to studying health care payment schemes that provides higher accessibility to the doctors through an online experiment with virtual diagnosis and prescription system. Furthermore, the results of this study implicate that what was considered a moral hazard may be a response to the health care system, which leaves a potential for future optimization. Our findings call for a consideration of the possible impact to the doctors’ behavior prior to the transition of health care payment system.
